# Recent Advances in Understanding and Managing Cardiomyopathy

**DOI:** 10.12688/f1000research.11669.1

**Published:** 2017-09-07

**Authors:** Paulino Alvarez, WH Wilson Tang

**Affiliations:** 1 Department of Cardiovascular Medicine, Heart and Vascular Institute , Cleveland Clinic, Cleveland, Ohio, USA; 2 Center for Clinical Genomics, Cleveland Clinic, Cleveland, Ohio, USA

**Keywords:** cardiomyopathy, heart transplant, cardiomyocytes

## Abstract

Cardiomyopathy is a disease of the heart muscle leading to abnormal structure or function in the absence of coronary artery disease, hypertension, or valvular or congenital heart disease. Currently, cardiomyopathy is the leading diagnosis of heart transplant patients worldwide. Incorporation of next-generation sequencing strategies will likely revolutionize genetic testing in cardiomyopathy. The use of patient-specific pluripotent stem cell-derived cardiomyocytes for disease modeling and therapeutic testing has opened a new avenue for precision medicine in cardiomyopathy. Stem cell therapy, gene therapy, interfering RNA, and small molecules are actively being evaluated in clinical trials.

## Introduction

Cardiomyopathy is a disease of the heart muscle leading to abnormal structure or function in the absence of coronary artery disease, hypertension, or valvular or congenital heart disease ^1^. Morphological subtypes include dilated cardiomyopathy, hypertrophic cardiomyopathy (HCM), arrhythmogenic right ventricular (RV) cardiomyopathy, left ventricular (LV) non-compaction, and restrictive cardiomyopathy. The global number of deaths and disability attributed to cardiomyopathy and myocarditis has steadily increased ^2, 3^. Cardiomyopathy represents the leading cause of cardiac transplantation ^4^. The hereditary nature of most cardiomyopathies creates an opportunity for early detection through family screening but also has the potential for misdiagnosis ^5^. How we classify, detect, and treat cardiomyopathy in 2017 and what is on the horizon are the focus of our review. In addition, infiltrative myocardial disease as an important differential diagnosis will be discussed when appropriate.

## Classification

As stated in the “Report of the 1995 World Health Organization/International Society and Federation of Cardiology Task Force on the Definition and Classification of Cardiomyopathies”, a classification is an attempt to “bridge the gap between ignorance and knowledge” ^6^. In that document, the following morphological and functional subtypes were recognized:

- Dilated cardiomyopathy: characterized by ventricular dilatation and impaired contraction
- HCM: characterized by LV and/or RV hypertrophy, which is usually asymmetric and involves the interventricular septum
- Restrictive cardiomyopathy: characterized by restrictive filling and reduced diastolic volume of either or both ventricles with normal or near-normal systolic function and wall thickness
- Arrhythmogenic RV cardiomyopathy: progressive fibro-fatty replacement of RV myocardium, initially with typical regional and later global right and some LV involvement, with relative sparing of the septum
- Unclassified cardiomyopathies: unclassified cardiomyopathies include a few cases that do not fit readily into any group

The 2006 American Heart Association (AHA) classification divided cardiomyopathies into primary, which are solely or predominantly confined to heart muscle, and secondary cardiomyopathies, which show pathological myocardial involvement as part of generalized systemic (multi-organ) disorders ^7^. Primary cardiomyopathies are subclassified into genetic, mixed (genetic and non-genetic), and acquired. In 2007, the European Society of Cardiology proposed a new classification in which each morpho-functional subtype was subclassified according to familial/genetic and non-familial/non-genetic forms. The familial/genetic forms were subdivided into unidentified gene defect and specific disease subtype, and the non-familial/non-genetic into idiopathic and specific disease subtype ^8^. “Specific cardiomyopathies” is used to describe cardiomyopathies associated with specific cardiac or systemic disorders. For example, a recent AHA document reviews diagnostic and treatment strategies for specific cardiomyopathies such as cardiac amyloidosis, cardiotoxins, peripartum cardiomyopathy, cardiac sarcoidosis, myocarditis, autoimmune cardiomyopathy, endocrine and metabolic cardiomyopathies, and genetic cardiomyopathies ^9^. Nevertheless, the application of morphological criteria can be misleading because diseases with similar imaging findings can have completely different pathophysiological mechanisms (for example, HCM and Fabry disease) ^10^.

**Figure 1.**
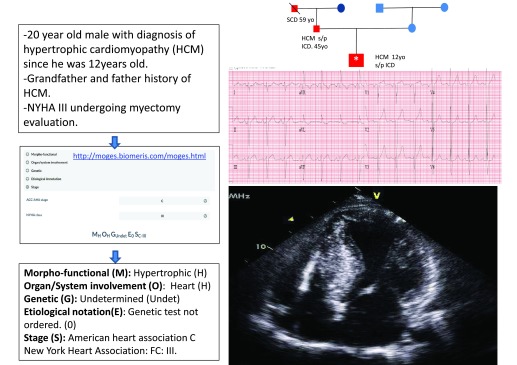
Example of MOGES classification utilization. Family diagram: square, male; circle, female; red, history of sudden cardiac death (SCD) or hypertrophic cardiomyopathy (HCM); blue, no history of SCD or HCM. Index case: *Age at diagnosis or SCD. Since no genotyping was performed, the mode of inheritance cannot be determined with certainty. FC, functional class; ICD, implantable cardioverter defibrillator; NYHA, New York Heart Association; s/p, status post.

The MOGE(S) classification of cardiomyopathies proposes a descriptive phenotype and genotype nosology system that incorporates the following five attributes: morpho-functional phenotype, organ(s) involvement, genetic inheritance pattern, etiological annotation including genetic defect or underlying disease/substrate, and functional status of the patient and disease process using both the American College of Cardiology/AHA stage and New York Heart Association functional class. The MOGE(S) nosology system is the most recently proposed ^1^. The use of a web-based application facilitates the implementation of this classification system ( http://moges.biomeris.com/moges.html) ( Figure 1).

Because the presence of a morphological abnormality is not synonymous with cardiomyopathy (for example, imaging criteria for LV non-compaction may be seen in more than 10% of persons free from cardiovascular disease), and the risk of developing genotype-positive and phenotype-negative cardiomyopathy is unknown, the clinician faces major challenges when evaluating an individual patient ^11, 12^. Quarta *et al*. recently proposed a framework to navigate this uncertainty ^13^. Their major criteria to diagnose cardiomyopathy include “1- Marked morphological abnormalities, clearly outside the limits of physiological remodeling, 2- Clear evidence of global or regional LV (and/or RV) systolic or diastolic dysfunction, or dynamic LV outflow obstruction at rest or on effort, 3- Frequent (>10,000 beats per 24 h) and repetitive ventricular ectopic beats, which do not subside or tend to increase with exercise and/or are polymorphic, or runs of sustained or nonsustained ventricular tachycardia, 4- Symptoms such as dyspnea, angina, pre-syncopal or syncopal episodes of non-vasovagal nature (particularly if upon or after exertion or after a meal), or reduced performance as assessed by cardiorespiratory testing, 5- A positive genetic test — when available and robust — or specific family history of cardiomyopathy and/or juvenile heart failure or sudden death; if unknown or not investigated, judgment should be suspended until relatives have been screened”.

## Detection of abnormal structure and function

### Non-invasive diagnostic modalities

Echocardiography is often the first diagnostic modality used when cardiomyopathy is suspected. Although morphological two-dimensional echocardiography and ejection fraction are key elements for phenotype characterization, advanced echocardiographic modalities such as tissue Doppler and strain have made possible the detection of early stages of myocardial dysfunction.

Abnormal tissue Doppler signals may help to identify the presence of HCM in the pre-hypertrophic stage and to differentiate athlete’s heart from HCM ^14, 15^. Machine learning models that incorporate longitudinal strain, among other volumetric and mechanical function variables, may help in the distinction between physiologic and pathologic hypertrophy ^16^. In patients with sarcoidosis but without evidence or history of cardiac involvement, impairment of global LV longitudinal strain was associated with increased risk of cardiac events ^17^. In patients exposed to anthracyclines, a relative percentage reduction in global longitudinal strain of at least 15% early during the course of therapy predicts the development of cardiotoxicity ^18^. Abnormal regional patterns of strain are useful for the identification of specific cardiomyopathies such as apical sparring usually seen in patients with preserved ejection fraction heart failure due to cardiac amyloidosis or apical compromise in patients with apical HCM ^19, 20^. In patients with obstructive HCM refractory to medical therapy who underwent septal myectomy, basal septal (myocardium removed during myectomy) mean systolic strain and diastolic strain rate correlated with *in vitro* measured myocardial contractile performance ^21^. Longitudinal strain and early diastolic strain pre-transplant showed significant correlations with mRNA expression of titin isoforms, sarcoplasmic reticulum Ca ^2+^ ATPase, and phosphorylated phospholamban in dilated cardiomyopathy ^22^.

Vector flow mapping, a novel method of Doppler signal analysis, has shown that the cause of systolic anterior motion of the mitral valve and obstruction in HCM is early systolic ejection flow or isovolumetric vortical flow impacting the posterior aspect of the mitral valve and not Venturi forces related to high flows in the LV outflow tract ^23^. Also, vortex formation has been correlated with functional capacity in a pilot study ^24^.

Cardiac magnetic resonance imaging has become an integral part of cardiomyopathy evaluation and risk stratification. Apical–basal bundles and mitral valve abnormalities (for example, increased anterior mitral valve length) have emerged as new phenotypic markers of HCM that are observed also in the pre-hypertrophic stage ^25, 26^. Fractal dimension, a parameter used to quantify myocardial muscle trabecular complexity, is increased in HCM mutation carriers without LV hypertrophy ^27^. LV structural abnormalities in patients with arrhythmogenic RV dysplasia have also been described with increased frequency ^28, 29^.

The presence, distribution, and burden of macroscopic myocardial scar tissue as measured by late gadolinium enhancement (LGE) are of particular importance. In a meta-analysis of 2,390 patients with various types of dilated cardiomyopathies, LGE was independently associated with higher risk of sudden cardiac death (SCD) and ventricular arrhythmias ^30^. In HCM, for every 10% increase in LGE, there was a 40% increase in SCD events. Of particular importance is that, in patients who were considered to be at low risk of SCD by traditional risk stratification systems, the presence of LGE of at least 15% of the myocardial mass had a five-year risk of SCD of 6% ^31^. In patients with Duchenne muscular dystrophy, the presence of LGE is associated with a progressive decline in LV ejection fraction (LVEF), and the magnitude of this decline is proportional to the number of myocardial segments with LGE ^32^. In patients with cardiac amyloidosis, cardiac magnetic resonance imaging shows a characteristic pattern of global subendocardial LGE; in addition, patients with systemic AL amyloidosis show markedly increased non-contrast T1 relaxation times in the myocardium when compared with healthy controls and patients with aortic stenosis ^33, 34^.

Extracellular volume fraction, which includes both diffuse and macroscopic patchy fibrosis using the modified look–locker inversion (MOLLI) recovery method, has been shown to correlate better with regional LV myocardial velocities than ejection fraction in patients with non-ischemic cardiomyopathy ^35^. Increased interstitial fibrosis is a marker of subclinical cardiac involvement in patients who are carriers of lamin A/C gene mutation ^36^.

Recently, *in vivo* diffusion tensor cardiac magnetic resonance has been shown to characterize the microstructural dynamics by analyzing sheetlet (laminar microstructures 5–10 cardiomyocyte thick) mobility and orientation during the cardiac cycle. Reduced mobility is seen in both dilated cardiomyopathy and HCM. Nevertheless, in HCM, abnormal diastolic conformation is present, whereas abnormal systolic conformation characterizes dilated cardiomyopathy ^37^.

The use of four-dimensional (4D) flow (3D + time = 4D) allows the generation of 3D streamlines that permit the analysis of different flow patterns. The application of this technique in patients with HCM, with and without obstruction, revealed abnormal flow patterns in the ascending aorta in both groups. The significance of these findings is unknown ^38^.

Cardiac positron emission tomography evaluates the presence of perfusion and metabolic abnormalities. In cardiac sarcoidosis, the coexistence of myocardial perfusion defects and increased focal metabolic activity, which reflects active disease, is associated with increased risk of death and ventricular tachycardia ^39^. ^99m^Technetium phosphate derivatives can bind to transthyretin in the myocardium and can be used to identify wild-type and mutant transthyretin-related amyloidosis. If amyloidosis is suspected and a scan is negative, this favors the diagnosis of light-chain amyloidosis ^40^.

### Invasive diagnostics

Invasive hemodynamic evaluation is particularly useful to differentiate restriction from constriction. In this case, simultaneous pressure measurements of the right and left ventricles are performed ideally with high-fidelity conductance catheters to evaluate for the presence of enhanced ventricular interaction and systolic area index in the case of constrictive pericarditis. Careful evaluation is necessary to avoid missing the diagnosis of a potentially reversible condition ^41^.

### Endomyocardial biopsy

Transvenous endomyocardial biopsy is an invasive procedure with a reported major complication rate (for example, tamponade) of less than 1% ^42^. The importance of tissue-based diagnosis for accurate prognostication in patients with unexplained cardiomyopathy has been known for a long time ^43^. The procedure is usually performed by using fluoroscopic guidance, with increasing interest in the use of 3D echocardiography to decrease the chances of RV free wall biopsy ^44^. The endomyocardial biopsy sensitivity can reach almost 100% in amyloidosis or approximately 30% in focal diseases such as sarcoidosis ^45, 46^. Usually, RV biopsy is performed, but the biventricular approach has been reported to increase diagnostic yield with a similar major complication rate in very experienced operators ^47^. The performance of voltage mapping to guide endomyocardial biopsy has been described to increase diagnostic yield in patients with focal myocardial diseases such as sarcoidosis or lymphocytic myocarditis ^48^.

In myocarditis, the use of immunohistochemistry to characterize inflammatory mechanisms, molecular techniques such as polymerase chain reaction to detect viral genomes, and—more recently—the analysis of non-coding transcripts such as microRNA hold the promise to continue to improve prognostication and treatment in this potentially lethal condition ^49, 50^. Interestingly, the presence of focal derangement or diffuse lysis of myofilaments observed by electron microscopy in the endomyocardial samples obtained from the posterolateral LV wall of patients with dilated cardiomyopathy admitted with acutely decompensated heart failure was a strong predictor of death or heart failure re-hospitalization during a follow-up period of 4.9 ± 3.9 years ^51^.

### Device therapy/roles of electrophysiology study/ablation

Implantable cardioverter defibrillators are indicated for the prevention of SCD in selected patients with heart failure and reduced ejection fraction and HCM at high risk of sudden death ^52, 53^. In cardiac sarcoidosis, the use of programmed electrical stimulation may assist in SCD risk stratification ^54^. Approximately 30% of patients will fail to respond to cardiac resynchronization therapy, and many causes or associated factors have been identified (for example, atrial fibrillation and less than 90% of biventricular pacing) ^55^. A novel mechanism to potentially improve cardiac resynchronization response is pacemaker-induced transient asynchrony. Six hours of daily RV pacing halted LV dilatation and myocyte dysfunction in a tachycardia-induced cardiomyopathy model in dogs ^56^. The recognition of a significant premature ventricular contraction burden (>24%) should increase the suspicion of premature ventricular contraction-induced cardiomyopathy; this is important because ablation of the ectopic foci may lead to improvement of cardiac function ^57^.

Electro-anatomic scar patterns differentiate RV outflow tachycardia from arrhythmogenic RV/dysplasia (ARVD) or cardiomyopathy. In ARVD, dominant sub-tricuspid scars with extensions toward the apex and RV outflow tract (RVOT) are seen where, in RVOT, isolated RVOT scar may be seen ^58^. The use of electro-anatomical mapping to guide endomyocardial biopsy has been previously described ^48^.

### Genetic evaluation and testing

Most cardiomyopathies are monogenic disorders. Unexplained cardiomyopathy, family history of SCD or cardiomyopathy, and electrocardiogram suggesting an inherited arrhythmia should trigger genetic evaluation ^59^.

The first fundamental step is a family history using a standardized template (for example, https://familyhistory.hhs.gov/FHH/html/index.html) if possible. Early consultation with genetics professionals is encouraged. It is important to convey the reason for consultation and potential implications to the patient. In cardiomyopathy, the main roles of genetic testing are to aid in the identification of family members at risk for the condition and to inform screening strategies.

In a large study of 312 patients with dilated cardiomyopathy, a truncating titin mutation was present in one-fourth of familial and in 18% of sporadic cases. Truncating titin mutations were observed in only 1% and 3% of patients with HCM (n = 231) and controls (n = 249), respectively ^60^.

Coppini *et al*. have reported that patients with HCM who have a mutation affecting the thin filament are at increased risk of developing LV dysfunction, heart failure, and severe diastolic dysfunction when compared with patients with thick filament mutations ^61^. Nevertheless, it is important to recognize that HCM is a disease where gene-phenotype correlations are more notable for their absence than presence.

Studies showing the impact of genotype in prognosis in ARVD/C have been reported. Patients with ARVD/C and carriers of a desmosomal gene mutation have worse outcomes when compared with those with titin mutations ^62^. In addition, in a large cohort of patients with ARVD/C who had pathogenic mutations, desmoplakin mutations were overrepresented in patients with SCD/ventricular fibrillation as a presenting symptom ^63^. In the same study, the negative prognostic implications of having more than one pathogenic mutation and male sex were also reported.

Future applications may include the development of genotype-specific therapies, and pilot studies of pharmacologic therapies in the pre-phenotype phase have been completed (see below). If concomitant congenital malformations are present and a chromosomal disorder is suspected, a chromosomal microarray analysis should be considered. When cardiomyopathy is the main feature and genetic abnormalities at the nucleotide level are suspected, several options are available. Gene panels directed to specific morpho-functional phenotypes that screen for pathogenic mutations are available. Results of genetic testing have five categories: pathogenic, likely pathogenic, variables of unknown significance, likely benign, and benign. The diagnostic yield of these panels is variable and ranges from 50 to 60% in ARVD and from 20 to 30% in dilated cardiomyopathy ^64^. Pan-cardiomyopathy panels have not been shown to increase the diagnostic sensitivity in HCM ^65^. The decrease in cost and turn-over time of next-generation sequencing strategies, including exome (sequences the genes involved in protein synthesis) and whole genome (sequences both coding and non-coding), has led some institutions to adopt this strategy. From a clinical standpoint, one of the main challenges of non-directed second-generation sequencing is the increase in the identification of variants of unknown significance; this is non-actionable genetic information ^66^. Nevertheless, recent reports combining these techniques with functional studies show encouraging results in detecting new pathogenic mutations ^67^. The creation of large repositories of genetic and phenotypic information, along with powerful analytic techniques, will shape the implementation of next-generation sequencing ^68^.

The incorporation of genetic information in epidemiologic studies with long-term follow-up has opened a new chapter in understanding the natural history of disease. Amyloidosis is a systemic disease due to the deposition of misfolded proteins that may affect the heart. There are two main forms of cardiac amyloid disease: AL amyloidosis due to immunoglobulin light chains and transthyretin amyloidosis. Patients with transthyretin may have wild-type (previously called senile amyloidosis) or genetic variants. The V122I variant, where isoleucine is substituted for valine, has a prevalence of approximately 3% in African-Americans and is the most common point mutation associated with hereditary transthyretin amyloidosis. Carriers of this mutation were believed to have an increased risk of death. In a study that included 3,856 African-Americans, 124 carriers were detected, and over a follow-up period of 21.5 years, those patients were at increased risk of heart failure development, but there were no statistically significant differences in mortality ^69^.

Other genetic variants include Val30Met (Any, Portuguese, Spanish) and Thr60Ala (Irish), both of which commonly affect the nerves and the heart. Nevertheless, early cardiac involvement is not as frequent in Val30Met ^70^.

### Precision medicine and emerging therapeutic strategies

Precision medicine is an emerging approach for disease treatment and prevention that takes into account individual variability (genes, environmental factors, and lifestyle) and is of particular relevance for cardiomyopathy ^71^. In this regard, the development of transcription factor-mediated reprogramming techniques, which allowed the production of human-induced pluripotent stem cells (hiPSCs), represents a major step forward ^72^. Protocols for the production of patient-specific pluripotent stem cell-derived cardiomyocytes (hiPSC-CMs) are available ^73^. Applications of hiPSC-CMs include disease modeling, regenerative medicine, drug discovery, and toxicity screening ^74^. For hiPSC-CMs that carry a mutation in cardiac transcription, factor TBX20 develops phenotypic characteristics of LV non-compaction cardiomyopathy. In this experiment, abnormal transforming growth factor-beta (TGF-β) signaling was detected. Inhibition of TGF-β signaling and genomic correction of the TBX20 mutation were sufficient to reverse the phenotype ^75^. Using patient-derived hiPSC-CMs, the mechanisms by which titin mutations cause sarcomere insufficiency in dilated cardiomyopathy were explored ^76^. In another exciting experiment, hiPSC-CMs from patients who experience doxorubicin cardiac toxicity were more sensitive to *in vitro* doxorubicin toxicity than hiPSC-CMs of patients who did not experience doxorubicin toxicity. Several mechanisms were identified, including decreased cell viability, impaired mitochondrial function, metabolic derangements, and increased production of reactive oxygen species ^77^. This finding suggests that hiPSC-CMs could be a potential tool to predict chemotherapy-induced cardiotoxicity.

Mutation silencing therapy in a murine model of HCM has shown that irreversible triggers to phenotypic expression occur early in development (<6 weeks of life) ^78^. One of the advantages of detecting genotype-positive phenotype-negative patients is the potential to intervene and alter the trajectory of disease. In a pilot and first-of-its-kind study, 38 patient carriers of pathogenic sarcomere mutations associated with HCM, but without LV hypertrophy, were randomly assigned to diltiazem or placebo. Patients who received diltiazem showed stable LV diameter and mean thickness-to-dimension ratio compared with control patients who showed a decrease and an increase in LV diameter and mean thickness-to-dimension ratio, respectively ^79^.

There has been pre-clinical evidence of the potential benefit of renin-angiotensin axis inhibition in modifying the course of HCM in a randomized controlled trial that included 133 patients with obstructive and non-obstructive cardiomyopathy, but the administration of losartan, though well tolerated, did not show significant difference when compared with placebo with regard to the primary endpoint (change in LV mass as measured by computed tomography or cardiac magnetic resonance imaging) ^80, 81^.

The presence of LGE has been used to identify patients with Duchenne and Becker muscular dystrophies who have normal LVEF but who are at higher risk of developing cardiac dysfunction. In this particular patient population, treatment with eplerenone (Duchenne) and angiotensin-converting enzyme inhibitor (Duchenne and Becker) delayed and attenuated adverse cardiac remodeling ^82, 83^.

The value of moderate exercise training in selected patients with HCM has been recently evaluated in a pilot randomized trial. After 16 weeks, patients randomly assigned to the unsupervised exercise protocol had a small but significant increase in exercise capacity as measured by cardiopulmonary exercise testing with no significant adverse events ^84^. In non-ischemic dilated cardiomyopathy, myocardial blood flow at rest and after cold pressor test improved after 12 weeks of cardiac rehabilitation ^85^.

### Target therapy/small molecules

MYK-461, a small molecule which binds to myosin decreasing its ATPase activity in a dose-dependent manner and consequently sarcomere contractility, has been shown to prevent the development of the HCM phenotype in a murine model of HCM if administered early (8–15 weeks of age) in the pre-hypertrophic stage, and it partially reversed structural abnormalities if administered once the hypertrophic phenotype became manifest ^86^. The intravenous administration of MYK-461 decreased contractility and eliminated systolic anterior motion of the mitral valve, relieving outflow obstruction in a feline model of HCM ^87^. Initial experience in humans shows encouraging results with a dose-dependent reduction of contractility and abolition of LV outflow gradients in two patients. Of note, one patient experienced asystole after receiving the highest dose studied, but it resolved without intervention ^88^.

Noonan syndrome and Noonan syndrome with multiple lentigines (formerly LEOPARD syndrome) as other RASopathies are autosomal dominant disorders caused by germline missense mutations of the Ras/mitogen-activated protein kinase (Ras/MAPK) signaling pathway; one of the phenotypic abnormalities is the development of HCM. A hyper-tyrosil phosphorylated form of protein-zero related (PZR) is present in the hearts of mice with those conditions. Yi *et al*. described that, in a murine model of Noonan syndrome and Noonan syndrome with lentigines, the administration of low-dose dasatinib (tyrosine kinase inhibitor) improves cardiomyocyte contractility, reduces fibrosis, and—if administered *in utero*—rescues HCM phenotype ^89^.

The *LMNA* gene encodes lamins C and A that are the major constituents of nuclear lamina, a proteinaceous meshwork that gives structural support to the nucleus and enables correct gene expression and DNA repair. Lamins A and C are generated through alternative splicing ^90^. Laminopathies are a diverse group of disorders caused by *LMNA* gene mutations and the most common one is dilated cardiomyopathy ^91^. Current pathophysiological mechanisms of *LMNA* mutations include increased susceptibility to mechanical stress, altered gene expression, and accumulation of pre-lamin A ^92, 93^. The feasibility of alternative splicing modulation with antisense oligonucleotides to increase lamin C and decrease pre-lamin A accumulation has been successfully tested in fibroblasts of patients with Hutchinson-Gilford progeria syndrome and a mouse model ^94^. The recognition of abnormal activation of the MAPK and Akt/mTOR (mammalian target of rapamycin) pathways has led to promising experimental data using protein kinase inhibitors and mTOR inhibitors, respectively, to block the development of cardiac dysfunction in mouse models of laminopathies ^95, 96^.

Constitutive activation of mammalian target of rapamycin complex 1 (MTOR-1) by tuberous sclerosis mutations is a recognized mechanism of tumor formation in this syndrome. In a patient with tuberous sclerosis and cardiomyopathy in which phosphorylation of ribosomal protein S6 (marker of MTOR-1 activation) was detected in endomyocardial biopsy, the administration of everolimus (MTOR inhibitor) led to improvement in systolic function and LV dimensions ^97^.

### Gene therapy in cardiomyopathy and heart failure

Increased understanding of the molecular mechanisms of heart failure have led to the recognition of potential targets for gene therapy ^98^. Increasing cardiac contractility by targeting molecules related to calcium cycling (sarcoplasmic reticulum Ca ^2+^ ATPase, or SERCA2a) or increasing beta-adrenergic system function (human adenylyl cyclase type 6, or hAC6) and enhancing stem cell tissue repair by increasing the expression of stem cell-derived factor 1 (SDF-1) have undergone clinical trial evaluation ^99^. Calcium upregulation by percutaneous administration of gene therapy in patients with cardiac disease (CUPID 2) that included 250 patients with NYHA (New York Heart Association) functional class II–IV and LVEF of not more than 35% who were randomly assigned to intracoronary infusion of adeno-associated virus loaded with SERCA gene versus placebo failed to achieve the primary endpoint of recurrent events (hospital admission because of heart failure or ambulatory treatment for worsening heart failure) with a median follow-up of 17.5 months. Nevertheless, the absence of significant adverse events is promising and has helped to enhance the interest in this therapeutic strategy ^100^. A recent phase II trial has shown a significant increase in LVEF in patients who received intracoronary delivery of adenovirus hAC6 when compared with placebo ^101^. **

### Cell therapy in non-ischemic cardiomyopathy

Intravenous allogenic mesenchymal stem cells were shown to be safe and effective in improving the 6-minute walk test and quality-of-life measures in patients with non-ischemic cardiomyopathy with a mean LVEF of 31%. There was no evidence of myocardial scar as measured by LGE in patients on maximal medical therapy ^102^. In a previous article, trans-endocardial injection of allogenic mesenchymal stem cells was superior to auto-mesenchymal stem cells in terms of efficacy (LVEF improvement and 6-minute walk test) and safety with an extremely low risk of allosensitization ^103^. A phase I clinical trial of transplantation of scaffold-free cell sheets derived from autologous muscle to the epicardial surface through left thoracotomy in patients with severe LV dysfunction due to ischemic (n = 15) or dilated cardiomyopathy (n = 12) has shown promising results regarding safety with no major procedural adverse events and efficacy with improvement in symptoms, 6-minute walk test, and LVEF ^104^.

### New developments in treating amyloidosis

Novel therapies that block abnormal protein synthesis such as small interfering RNA and antisense oligonucleotides are undergoing phase III clinical trial evaluation in transthyretin amyloidosis, the most common form of cardiac amyloidosis ^70^. In light-chain amyloidosis, the use of an amyloid fibril-reactive chimeric monoclonal antibody is undergoing clinical trial evaluation. This approach was shown to be safe in a phase I clinical trial that included six patients with refractory amyloidosis. Those three patients showed organ response: two cardiac and one gastrointestinal ^105^.

### Immune modulation in heart failure

Inflammation is a key player in the development and progression of heart failure ^106^. Anti- tumor necrosis factor therapy has been shown to be ineffective and even potentially harmful in this patient population in large randomized clinical trials ^107^. Non-specific immunomodulation was also unsuccessful in reducing death from any cause and hospitalization from cardiovascular causes ^108^. There is increasing interest in the manipulation of the innate immune system ^109^. Toll-like receptors, which are the primary receptors of the innate immune system and related molecules (for example, myeloid differentiation 1), constitute attractive targets that are the subject of intense research ^110, 111^. In addition, the recognition that embryonic-derived macrophages that have anti-inflammatory properties and promote tissue regeneration are replaced in heart failure with monocyte-derived macrophages that have pro-inflammatory properties may open a new pathway to cardiac recovery ^112^.

### New surgical approaches

In HCM, surgical septal myectomy often resolves mitral regurgitation related to systolic anterior motion of the mitral valve; nevertheless, some patients need additional mitral valve procedures such as mitral valve repair or replacement ^113^. A novel operative technique that consists of trans-aortic cutting of thickened secondary mitral valve chordae seems to be effective in relieving outflow tract obstruction in patients with HCM and mild septal thickness with the advantage of avoiding additional mitral valve procedures ^114^. This technique is based on the pathophysiological hypothesis that fibrotic and retracted secondary chordae may cause abnormal tethering of the anterior mitral valve and favor the displacement of the “slack portions of the leaflet (and attached primary chordae) into the LV outflow tract” ^114^.

## Conclusions

The number of patients affected by cardiomyopathies is increasing. Given the strong genetic basis of many cardiomyopathies, a complete family history is mandatory. Judicious use of cardiac imaging is extremely useful in defining the morpho-functional phenotype, informing prognosis, and detecting subclinical disease. Genetic testing is being increasingly incorporated into clinical practice. MOGES classification provides a good framework to facilitate communication and patient classification. The use of patient-specific pluripotent stem cell-derived cardiomyocytes for disease modeling and therapeutic testing is exciting and hopefully will be incorporated into clinical practice in the near future. Gene therapy, small molecules, small interfering RNA, and antisense oligonucleotides are being tested in clinical trials.
